# Two Cases of Lung Cancer in Foundry Workers

**DOI:** 10.1186/2052-4374-25-16

**Published:** 2013-09-16

**Authors:** Inchul Jeong, Innshil Ryu, Boowook Kim, Inhyo Park, Jong-Uk Won, Eun-A Kim, Inah Kim, Jaehoon Roh

**Affiliations:** 1 Graduate School of Public Health, Yonsei University, Seoul, Korea; 2 Institute for Occupational Health, Yonsei University College of Medicine, Seoul, Korea; 3 Korean Industrial Health Association, Suwon, Korea; 4 Occupational Lung Diseases Institute, Korea Workers’ Compensation and Welfare Service, Ansan, Korea; 5 Incheon Workers’ Health Center, Incheon Business Center, 636 Gojan-dong, Namdong-gu, Incheon, Korea; 6 Department of Preventive Medicine, Yonsei University College of Medicine, Seoul, Korea; 7 Occupational Safety and Health Research Institute, Korea Occupational Safety and Health Agency, Incheon, Korea

**Keywords:** Crystalline silica, Iron and steel founding, Lung cancer, Occupational exposure

## Abstract

**Background:**

Iron and steel foundry workers are exposed to various toxic and carcinogenic substances including crystalline silica, polycyclic aromatic hydrocarbons, and arsenic. Studies have been conducted on lung cancer in iron and steel founding workers and the concentration of crystalline silica in foundries; however, the concentration of crystalline silica and cases of lung cancer in a single foundry has never been reported in Korea. Therefore, the authors report two cases of lung cancer and concentration of crystalline silica by the X-ray diffraction method.

**Case presentation:**

A 55-year-old blasting and grinding worker who worked in a foundry for 33 years was diagnosed with lung cancer. Another 64-year-old forklift driver who worked in foundries for 39 years was also diagnosed with lung cancer. Shot blast operatives were exposed to the highest level of respirable quartz (0.412 mg/m^3^), and a forklift driver was exposed to 0.223 mg/m^3^.

**Conclusions:**

The lung cancer of the two workers is very likely due to occupationally related exposure given their occupational history, the level of exposure to crystalline silica, and epidemiologic evidence. Further studies on the concentration of crystalline silica in foundries and techniques to reduce the crystalline silica concentration are required.

## Background

Foundry workers are known to be exposed to various kinds of hazardous factors, and the iron and steel founding industry is classified into group 1 carcinogens by the International Agency for Research on Cancer (IARC) [1]. Previous epidemiologic surveys in Korea have reported that foundry workers are exposed to carcinogens like crystalline silica, polycyclic aromatic hydrocarbons, and arsenics [2]. Among the carcinogens, crystalline silica is a major risk factor for lung cancer.

Many cases of lung cancer in foundry workers have been reported in Korea [3], and an increased incidence of lung cancer and mortality by lung cancer have also been described in previous studies [4-6]. Other studies have reported that the concentration of crystalline silica exceeds the threshold limit value (TLV) of 0.025 mg/m^3^ by the American Conference of Governmental Industrial Hygienists (ACGIH). In one study in Korea, the mean concentration of crystalline silica in foundries was 0.027 mg/m^3^ and 34.0% exceeded the ACGIH-TLV [7]. Other studies have described that the concentration of crystalline silica in foundries to be 0.028 mg/m^3^ in Sweden and 0.082 mg/m^3^ in the U.S. [8,9].

Despite these reports, to the best of the authors’ knowledge, the concentration of crystalline silica of a foundry at which a work-related lung cancer patient worked has not been reported in Korea. Moreover, most of the studies in Korea have used the Fourier transform infrared (FTIR) spectroscopy method, which yields less precise results than the X-ray diffraction (XRD) method, and thus comparison of the results between studies is difficult [7-9]. Therefore, the authors report here two cases of lung cancer in a manufacturing worker and a forklift truck driver of a foundry, along with the exposure concentration of crystalline silica according to the manufacturing process by the XRD method.

## Case presentation

### Case 1

A 55-year-old man was diagnosed with non-small cell lung cancer in 2009. A lung biopsy showed adenocarcinoma, and additional tests (bone scan, spine MRI, PET-CT, lymph node biopsy) showed multiple bone, liver, and lymph node metastases (T3N3M1). He died 30 months after the initial diagnosis during chemotherapy.

He had no medical history other than hypertension, and the health insurance records showed no respiratory problems other than bronchitis and upper respiratory infection. Special health examination records also showed no abnormality other than hypertension. He was a non-smoker and social drinker, and enjoyed mountain climbing during his leisure time. His mother was diagnosed with lung cancer. He had no notable experience during his military service.

He worked at a foundry in Incheon, Korea beginning in 1976. From 1976 to 1993, he worked in the molding process, and worked in the shot blasting and grinding process from 1994. He was a day-shift worker and worked from 8 AM to 6 PM. He had never worked at another company.

### Case 2

**Figure 1:**
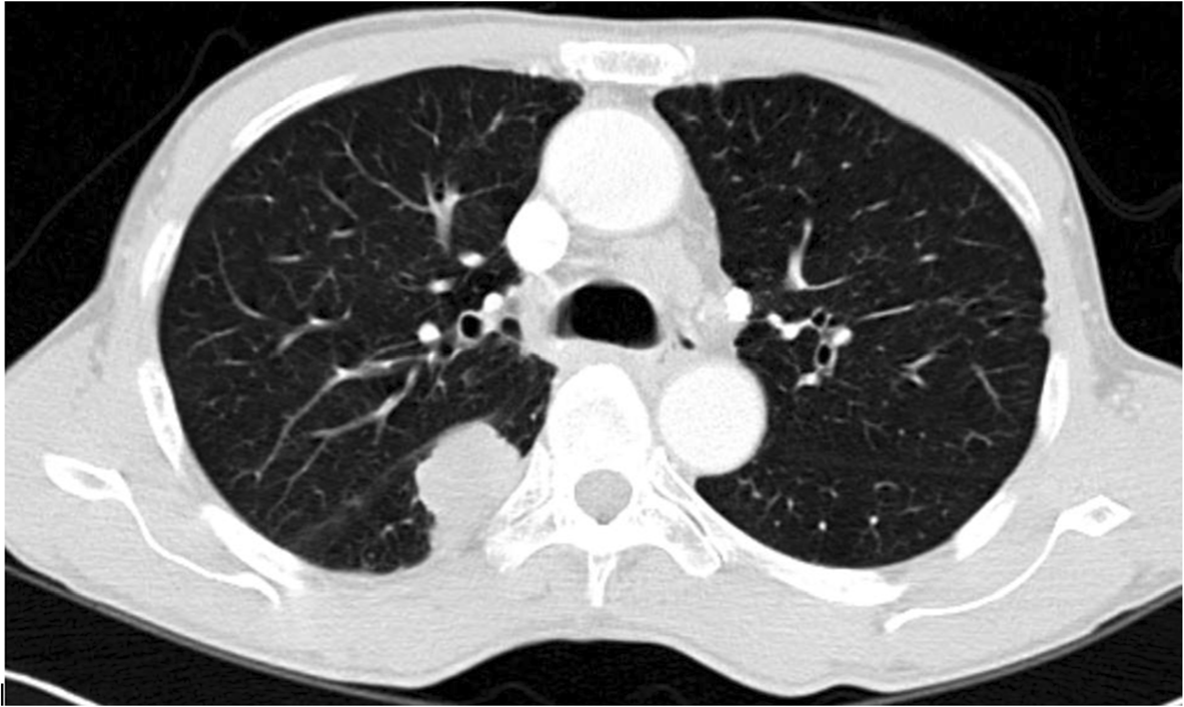
A contrast-enhanced chest CT shows a nodular mass in the superior segment of the right lower lobe.

A 64-year-old man was diagnosed with non-small cell lung cancer in 2010. In the general health examination, the chest X-ray result was ‘not yet diagnosed’ and pulmonary tuberculosis was diagnosed in the reexamination. He was treated with tuberculosis medications for 6 months; however, his symptoms were aggravated. He was transferred to another hospital and a chest CT and lung biopsy showed non-small cell lung cancer (T3N3M0, Figure 1). He died 15 months after presenting with the initial abnormality during chemotherapy and radiation therapy.

He had a history of hypertension, and his general health examination records showed cardiomegaly and arrhythmia. His special health examination records showed no abnormality; however, he had not been able to receive special health examinations when he had been employed as a subcontractor. He had a 35- to 40-pack-year history of smoking and consumed approximately 70 g of alcohol 3 to 5 times a week. He had no specific family history or notable experience during his military service.

He started forklift truck driving in 1971 and worked at another company until 1987. In 1987, he was employed at the foundry. From 1987 to 2001, his task was to move primary products (products before the shake-out process) or secondary products (products after the shake-out process) to the next process, and mainly worked around the areas of shot blasting and grinding. From 2001, his task changed to moving waste sands from the foundry to a disposal facility. He retired in 2004; however, was employed at a sub-tier supplier with the same task. His work schedule was rotating 12-hour shift.

### Outline of the company

**Figure 2:**
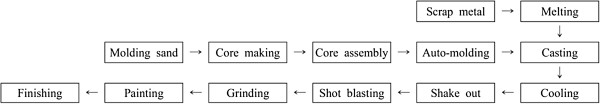
Process chart of a foundry.

The foundry, established in 1975, produces automobile engine parts. It was spun off in 2001 and is a subcontractor now. There are approximately 270 employees. The sub-tier supplier, where the patient of case 2 worked after retirement, provides manpower to the subcontractor. The sub-tier supplier employs approximately 50 manufacturing workers and forklift truck drivers. The patient of case 1 mainly worked at the shot blasting and grinding area. Shot blasting and grinding are the processes of removing the remaining sands using hammers and grinders after the cast has been removed. The manufacturing process of the foundry is described in Figure 2.

### Materials safety data sheet and working environment measurement results

According to the materials safety data sheet, the molding sand contains 35-40% quartz. Previous working environment measurements found that, from 2004 to 2010, the concentration of mineral dust consistently exceeded the Korean Ministry of Employment and Labor (MOEL) TLV (2 mg/m^3^ and 5 mg/m^3^ for mineral dust containing crystalline silica ≥ 30% and <30%, respectively), and the highest concentration level was 47.8 mg/m^3^. Until 2010, the gravimetric method was used to determine mineral dust concentrations, and FTIR spectroscopy was used to determine respirable quartz concentrations. In 2011, the mean concentration was 0.03 mg/m^3^, and the highest concentration was 0.54 mg/m^3^.

### Exposure assessments of crystalline silica and heavy metals

Exposure measurements of crystalline silica and heavy metals were performed in April 2012, targeting the processes at which the two patients worked (shot blasting, grinding, auto-molding, and forklift driving). Personal sampling of crystalline silica and heavy metals was performed for 2 shot blasting workers, 2 grinding workers, an auto-molding worker, and a forklift truck driver. The sampling time was 6 hours and exposure was calculated as an 8-hour time weighted average (TWA) concentration.

The concentration of crystalline silica was determined by the National Institute for Occupational Safety and Health (NIOSH) 7500 method [10]. Using a GS-1 cyclone (SKC, USA), respirable dust was collected on a polyvinyl chloride filter (37 mm, 5 um). After high temperature ashing, the residue was redeposited on a silver filter (25 mm, 0.45 um), then analyzed by XRD (D8 Advance, Bruker, Germany). The concentration of heavy metals was determined by the NIOSH 7300 method [10]. Total dust was collected on a mixed cellulose membrane filter (37 mm, 0.8 um) and processed with acid. It was then analyzed by inductively coupled plasma mass spectrometry (DRC-e, Perkin Elmer, USA).

The concentration of crystalline silica exceeded the MOEL-TLV of 0.05 mg/m^3^ by at least three times in all of the samples. The concentration of the sample from the shot blasting worker was 0.412 mg/m^3^, the highest among all of the samples, and the concentration of the sample from the forklift driver was also high (0.223 mg/m^3^). Moreover, cristobalite was found in the samples from the shot blasting worker and auto molding worker. However, in the case of heavy metals, no sample exceeded the MOEL-TLV of chromium, manganese, and iron. Nickel, arsenic, and cadmium were not detected in any of the samples (Table 1).

**Table 1:** Exposure assessments of crystalline silica, chrome, manganese, and iron

	**Crystalline silica (mg/m**^**3**^**)**		**Chromium (mg/m**^**3**^**)**	**Manganese (mg/m**^**3**^**)**	**Iron (mg/m**^**3**^**)**
	**Respirable quartz**	**Respirable cristobalite**			
Shot blast 1	0.412	<LOD			
Shot blast 2	0.153				
Grinding (fettling) 1	0.161		0.007	0.013	0.995
Grinding (fettling) 2	0.281		0.013	0.003	0.105
Auto-molding	0.250	<LOD	0.005	0.003	0.103
Forklift truck	0.223		0.006	0.007	0.608

LOD: limit of detection.

## Discussion

Foundry workers are exposed to various kinds of lung carcinogens. Crystalline silica, asbestos, formaldehyde, chromium, nickel, cadmium, and polycyclic aromatic hydrocarbons including benzo(a)pyerene, which workers can be exposed to, are classified as group 1 carcinogens by the IARC [1]. The patients reported in this paper were a worker who worked in the molding and surface cleaning process, which are the main manufacturing processes of a foundry, and a forklift truck driver, who was indirectly exposed to the hazardous factors. However, in all of the industrial processes tested, the concentration of crystalline silica exceeded the MOEL-TLV, as well as the ACGIH-TLV.

There are several suggested mechanisms by which crystalline silica induces lung cancer. First, crystalline silica and quartz-exposed macrophages and epithelial cells generate reactive oxygen species, which directly damage DNA. Second, quartz depletes antioxidant defenses in the lung and enhances oxidative damage. Third, inhaled crystalline silica injures alveolar macrophages and impairs clearance, thereby increasing the secretion of chemokines and cytokines [1].

A number of studies have investigated the effect of foundry working on lung cancer. In a study conducted in Korea, the standardized incidence ratio (SIR) of lung cancer was 1.45 (95% confidence interval (CI) 1.11-1.87) among foundry workers compared to the normal population, and the SIR (95% CI 1.09-2.12) was 1.55 among those who worked more than 10 years. In addition, the standardized rate ratio of lung cancer was 3.06 (95% CI 1.22-7.64) among molders and core makers, and 2.63 (95% CI 1.01-6.84) among fettlers compared to office workers [4]. In studies targeting foundry workers and lung cancer, the standardized mortality ratio (SMR) of lung cancer was 163.9 (95% CI 123.9-223.0) compared to the normal population in Germany, the odds ratio for death was 1.66 (95% CI = 1.16-2.38), the SMR was increased (SMR 3.98, 95% CI 1.41-16.6 among 4th quartile of exposure compared to 1st quartile) in the U.S., and the proportional mortality ratio was 1.2 (95% CI = 1.0-1.6) in China [5,6,11,12].

The concentration of respirable quartz in this company was 0.153-0.412 mg/m^3^, which is not greatly different from the highest concentration of the 2011 working environment measurement result of 0.542 mg/m^3^. This is 3 to 8 times the value of the MOEL-TLV and 6 to 16 times the value of the ACGIH-TLV, which is higher than the previously reported concentration in Korea. In two studies in Korea, the concentration of crystalline silica in foundries was 0.022-0.046 mg/m^3^ and 0.027-0.043 mg/m^3^[7,13]. One possible explanation is that most of the previous studies were conducted in foundries with less than 100 workers, while the number of workers in the company reported in this study was approximately 500 (including the number of workers at the sub-tier suppliers), and the products manufactured by the company are relatively large in size; therefore, a considerable amount of molding sand might be used. Another explanation is that due to the inappropriate control of dust, a large amount of old waste sand had piled up in the workplace and could easily be scattered. Further, there were problems in the local ventilation system; for example, the workers work between the source of dust and the ventilation ducts, and fans directed at the workers’ faces. In case of the forklift truck, the risk of exposure was present because there was a great deal of dust inside. Furthermore, there were no ventilators in the disposal facility.

Cristobalite was detected in the shot blasting and auto molding process. Cristobalite is formed from crystalline silica by phase transition and is known to have higher toxicity than quartz. There was a possibility of higher exposure in the 1970s to 1990s than in the 2000s, based on the workers’ comment that the working environment was poorer in the past and they started to wear personal protective equipment beginning in the mid-1980s. In addition, there was a possibility of asbestos exposure. According to the co-workers of the patients, they used a cover suspected to have been made out of asbestos to cover the furnaces. Though the authors could not identify any asbestos-related materials, the possibility of asbestos exposure cannot be fully excluded because there are reports of asbestosis, asbestos-related radiologic changes, and an increased SMR for mesothelioma in foundry workers [14-16].

In this case report, the XRD method was utilized to analyze crystalline silica, which is a most widely used method internationally. Although the FTIR spectroscopy method is most widely used in Korea, it is more prone to interference and difficult to detect cristobalite [17]. The measurement was performed in the processes of shot blasting, grinding, auto molding, and transporting, and the results for the manufacturing processes and transportation were similar, which is consistent with a previous study [8].

There are some limitations in this study. First, the working environment measurements were performed only in one company, and number of workers sampled was 6, which is not large enough to generalize the results. Second, whether the concentrations measured in this study represent the usual concentrations of the company is not certain because the amount of output at the time is not clearly understood. However, the result is reliable because the measurement was thoroughly performed under the supervision of an industrial hygienist. The strength of this study is that the exposure concentration of the forklift truck driver was compared to that of workers of other processes, including sub-tier supplier workers.

## Conclusions

In conclusion, the patients’ lung cancer was likely due to occupationally related exposure including crystalline silica. Therefore, a specific occupational health management system is required for industries with sufficient evidence of carcinogenicity. Strategies for health care to indirectly exposed workers like forklift truck drivers are also required, as the workers are excluded from special health examinations and working environment measurements. In addition, large-scale studies on the concentration of crystalline silica in foundries and techniques to reduce the crystalline silica concentration are required.

## Consent

Written informed consent was obtained from the patient for the publication of this report and any accompanying images.

## Competing interests

There is no competing interest or financial support to declare.

## Authors’ contributions

JI and KI designed the research. JI, RI, KB, and PI collected the data. KB and PI analyzed the data. JI, RI, WJU, KEA, RJ, and KI interpreted the data. JI and KI wrote the manuscript. All authors read and approved the final manuscript.
